# In Regard to Bozkurt et al.

**DOI:** 10.4274/balkanmedj.2018.0407

**Published:** 2018-07-24

**Authors:** Mete Özkıdık, Anar İbrahimov

**Affiliations:** 1 Clinic of Urology, Şanlıurfa Training and Research Hospital, Şanlıurfa, Turkey; 2 Department of Urology, Ankara University School of Medicine, İbni Sina Hospital, Ankara, Turkey

To the Editor,

I read the article by Bozkurt and Şahin (1) with great interest. I have some points of concern related to this case report.

First, the authors have stated that painful erections lasted for about 1 hours after adding olanzapine 2.5 mg/day. However, there was no comment regarding the number of attacks or the time of attacks. I believe that it is essential to determine whether periods of priapism occur during the day or the night in a 9-year-old boy because of possible physiological nocturnal erections in the peripubertal interval.

Second, in this case, priapism attacks were not observed by a health professional. Pain during erections or the color of the penis may lead us to a diagnosis of priapism; however, I believe that it should be clarified by an observation in the hospital. In addition, there are case reports of priapism with methylphenidate treatment (2,3). It would be safer to observe the patient for a few hours after the initiation of methylphenidate in case of recurrence of priapism.

Finally, the authors have stated that other organic causes of priapism were excluded. However, the details of the examination were not mentioned in the article. I believe that the details of any radiological evaluation performed should be mentioned. This is because the diagnosis and treatment of priapism are not very clear for this age interval and there are only a few guidelines in the pediatric urology literature (4).
